# Deriving a set of representative optical coefficients from the literature: an illustrative statistical framework for Monte Carlo simulations applied to the human skin

**DOI:** 10.1117/1.JBO.31.11.115001

**Published:** 2026-11-09

**Authors:** Emmanuel Gerelli, Georges Wagnières, Jaroslava Joniová

**Affiliations:** Swiss Federal Institute of Technology (EPFL), Institute of Physics, Laboratory for Functional and Metabolic Imaging, Lausanne, Switzerland

**Keywords:** skin optical properties, Monte Carlo simulation, absorption coefficient, scattering coefficient, anisotropy factor, refractive index

## Abstract

**Significance:**

Accurate characterization of light propagation in biological tissues is essential for applications such as phototherapy, photodermatology, photobiomodulation, and photodiagnosis. Because many organs are stratified, Monte Carlo simulations require sets of optical coefficients–absorption ($\mu_{a}$), reduced scattering ($\mu_{s}^{\prime }$), anisotropy factor (g), and refractive index (n)–in addition to tissue geometry, to model light transport in each tissue layer. However, published values of these coefficients often differ significantly for a given tissue and wavelength.

**Aim:**

This work aims to address the challenge of selecting representative optical coefficients for Monte Carlo simulations by compiling and critically evaluating reported optical properties of human skin (Phototype III) and muscle as an illustrative example.

**Approach:**

Published optical coefficients reported for selected visible and near-infrared wavelengths were analyzed and compared. A statistical framework was applied to derive representative sets of optical coefficients for different skin layers from heterogeneous literature data.

**Results:**

The analysis revealed substantial variability among reported optical coefficients. Representative sets of absorption, reduced scattering, anisotropy, and refractive-index values were derived for distinct skin layers and muscle at selected wavelengths.

**Conclusions:**

The proposed framework provides a practical approach for deriving representative optical coefficients from heterogeneous literature data and highlights the importance of standardized, multilayered tissue models for accurate and reproducible light-dosimetry simulations.

## 1 Introduction

The precise determination of light fluence rate F and, consequently, the fluence Ψ in biological tissues presents a significant challenge, especially when illumination and sample geometries are complex. To overcome analytical limitations, Monte Carlo (MC) computer-based simulations of light propagation have become indispensable tools in biomedical optics.^1^ These simulations require comprehensive knowledge of the sample’s illumination geometry, alongside its intrinsic optical properties such as the absorption coefficient $\mu_{a}$, the scattering coefficient $\mu_{s}$, the scattering anisotropy factor g, and the refractive index n.

As an illustrative example, a specific objective in photodermatology is to accurately predict the spatial distribution of the fluence rate within different layers of the human skin illuminated at various wavelengths. This necessitates a thorough understanding and compilation of the literature pertaining to the optical coefficients of human skin at relevant wavelengths. It is important to acknowledge the broad spectrum of theoretical and experimental methods reported for assessing these optical coefficients. Furthermore, these coefficients exhibit considerable dispersions, notably for values measured *in vivo*^1^ and, to a lesser extent, *ex vivo*.^2^ These disparities are primarily attributable to two main factors: a) variations in skin and tissue types, and b) differences in measurement methodologies, each with their respective biases.

Approaches used to determine tissue optical coefficients can generally be classified into two main groups: 1) analytical methods based on the biochemical composition of specific tissue layers, and 2) experimental measurements performed on tissues *in vivo* or *ex vivo*. Analytical models frequently derive optical properties from a baseline absorption and scattering, augmented by the relative contributions of the layer’s primary absorbers and scatterers.^3^ The accuracy of this method is contingent upon the estimated concentrations of the chromophores and scatterers considered. *Ex vivo* experimental measurements typically involve the careful separation of skin tissue layers, followed by their placement between integrating spheres.^4^ The measured transmittance and reflectance are then frequently utilized to calculate, often through inverse MC methods, the absorption and reduced scattering coefficients. The method of layer separation, storage conditions and duration, the time elapsed between excision and measurement, the specific measurement technique, the inverse problem-solving algorithm, and sample preparation all contribute to the relatively large variability observed in reported optical properties.^1^^,^^3^^,^^5^ This variability makes the identification of a unique set of optical parameters difficult for specific tissue layers and wavelengths. This situation is problematic since MC simulations require the use of a unique set of these parameters to generate “representative” or “consensus” results. This unique set of parameters is usually determined by simply averaging these coefficients, an approach that often overestimates the uncertainties in the resulting means, as discussed below.^6^^,^^7^ Consequently, the values presented in this review are not only derived from a critical interpretation and analysis of the existing literature, informed by extensive experience in measuring tissue optical properties,^4^^,^^5^^,^^8^^–^^22^ but are also proposing a new approach to derive unique sets of representative optical coefficients for distinct skin layers and subcutaneous muscle, as an illustrative example. In addition to optical properties, this report also addresses the geometric features of the skin’s histological layers. This is of paramount importance because these layers can exhibit significant optical differences, which profoundly impact the values of the fluence rate, particularly within the first few hundred microns below the air-skin interface. Finally, another goal of this report was to find typical values for epidermis, dermis, and fat at many wavelengths generally emitted by diodes in photo-treatments, including a device such as the ATP38® [Swiss Bio Inov, Moudon, Switzerland].

## 2 Skin Anatomy and the Biophysical Basis of Optical Properties

The skin, akin to most mucous membranes, is a complex heterogeneous medium characterized by cellular structures^23^ and chromophore concentrations that are highly variable with depth.^24^ Due to the intricate nature of these tissues and the substantial differences in optical properties between their constituent layers, a simplistic single-layer model assuming homogeneous tissue is generally inadequate for describing light propagation with reasonable precision.^6^^,^^22^^,^^25^^,^^26^ However, it is possible to define anatomical skin layers within which cellular structure and chromophore concentrations are considered as approximately homogeneous.^6^ Based on this observation, the skin can be broadly divided into three primary layers, as commonly depicted in anatomical models: 1) the epidermis (EPI), a thin, avascular layer; 2) the dermis (DER), a highly vascularized layer; and 3) the subcutaneous fat layer (FAT), an adipose tissue layer. These layers are illustrated in Fig. 1. The layer-thickness ranges shown in Fig. 1 encompass the various values reported in the following references.^1^^,^^2^^,^^6^^,^^22^^–^^24^^,^^27^ The authors acknowledge that the complexity of skin-layer thicknesses, together with inter- and intra-patient temporal and spatial variations, can affect the outcomes of Monte Carlo simulations.

**Fig. 1:**
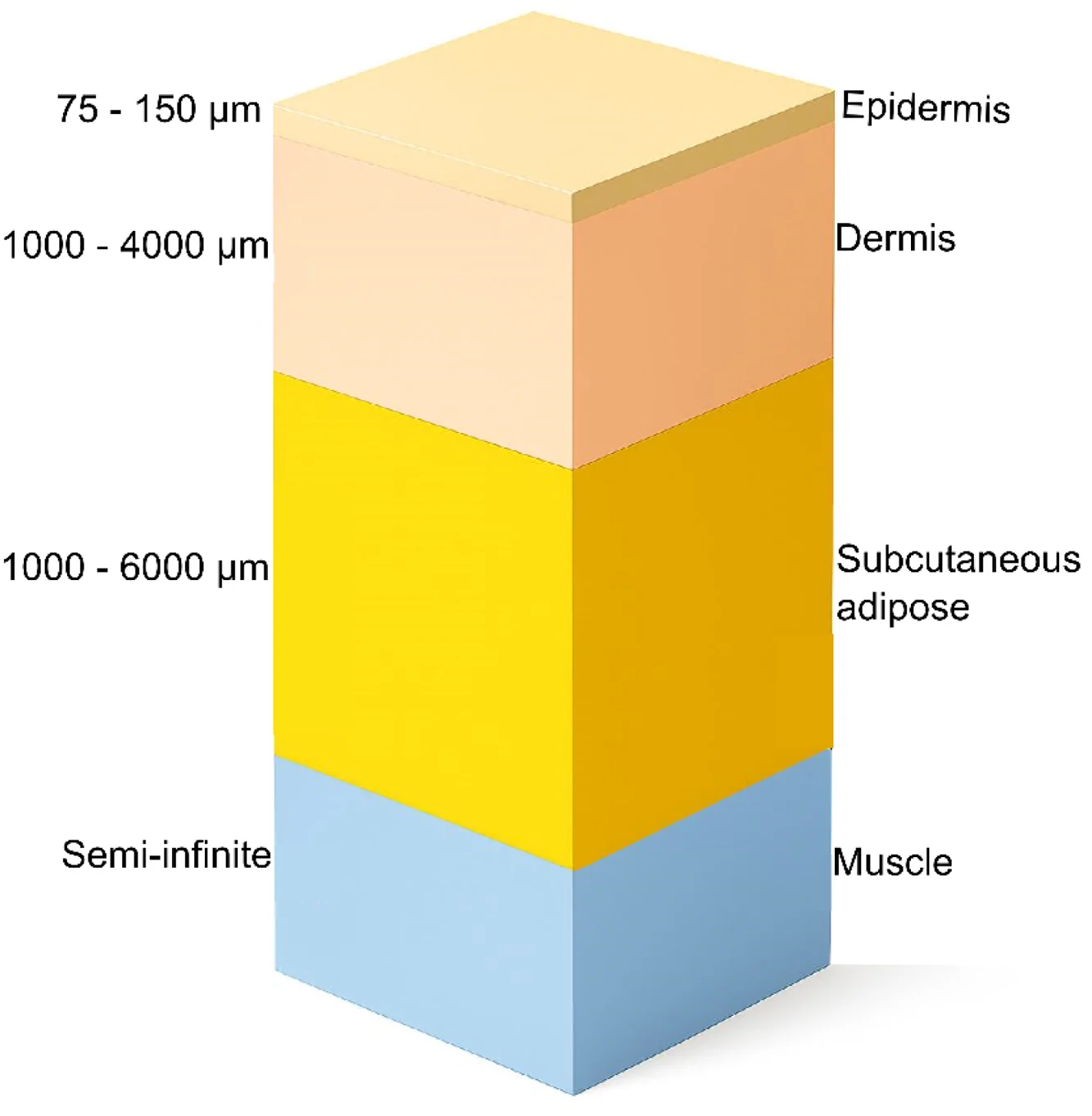
Schematic of the multilayered tissue model. The illustrative model, used to evaluate the fluence F with depth, is composed of four homogeneous layers: the epidermis, dermis, subcutaneous adipose,and an infinitely thick muscle layer.

Although these layers comprise subparts and other intricate structures, they are frequently approximated as bulk materials with homogeneous optical properties for modeling purposes. This segmentation not only provides a good description of *in vivo* conditions but also facilitates the quantification of skin optical properties through measurement. Consequently, these three skin layers have been the most extensively studied in the literature.^6^ It should be noted that the stratum corneum, due to its small thickness (typically between 10 and 40 microns), is rarely considered in light propagation modeling.^6^ This is mostly due to the fact that the reported optical coefficients of the stratum corneum, in particular, its refractive index,^27^ do not differ dramatically from those of the epidermis in the visible part of the spectrum. As a consequence, the impact of this layer on the propagation of light is minimal considering its small thickness.^6^ It should be noted that the thinness of the stratum corneum makes the experimental determination of its optical coefficients difficult.

### 2.1 Epidermal Optical Properties: Role of Melanin and Melanosomes

The epidermis is the primary site of skin pigmentation, predominantly due to the melanin contained within melanosomes. Melanin is the main absorber in this layer across the UV, visible, and NIR spectral ranges. The volume fraction of melanosomes within the epidermis varies significantly, ranging from approximately 1.3% in light-skinned Caucasians to as high as 43% in darkly pigmented Africans.^26^ Optical properties of the skin reported in the literature are predominantly for average Caucasian skin types (Phototype III), typically assumed to have a 5% melanosome volume fraction.^23^ Melanosomes also contribute to scattering due to their size and their substantial refractive index difference compared with their surrounding medium. It should be noted that the relative proportions of Eumelanin and Pheomelanin vary significantly for different skin types. These biomolecules feature broad, structureless absorption in the UV and visible part of the spectrum that increases toward shorter wavelengths.^28^ Eumelanin (black/brown) absorbs strongly across the UV and visible range with a smooth curve, whereas pheomelanin (reddish-brown) exhibits a higher molar absorptivity in the UV and near-UV range and distinct transient excited-state signatures. Melanosomes also exhibit changes of their sizes, shapes, and aggregation states, which change the epidermis optical properties. This co-presence of potent absorbers and scatterers explains why $\mu_{a}$ and $\mu_{s}^{\prime }$ values in the epidermis are approximately an order of magnitude larger than those found in the dermis and subcutaneous adipose tissue. The profound influence of these large coefficients on light propagation within the skin is counterbalanced by the relatively small thickness of the epidermis compared to deeper layers, even though this thickness exhibits a high variability. Indeed, epidermal thickness can range from about 660  μm on the soles of the feet to approximately 100  μm in the eyelids.^1^ It is also evident that epidermal optical properties are influenced by factors such as age, gender, and exposure to sunlight.^5^

### 2.2 Dermal Optical Properties: Role of Hemoglobin and Fibrous Structures

The dermis is a fibrous structure primarily composed of collagen, elastic tissue, and other extracellular components, providing support and protection to the skin and underlying layers.^29^ As a highly vascularized layer, its main absorber in the visible spectral range is due to the presence of hemoglobin, in addition to minor contributions resulting from carotene and bilirubin. Consequently, characteristic absorption peaks of hemoglobin (around 420 and 540 nm) are expected in the measured absorption spectrum of the dermis, while blood absorption significantly decreases above 630 nm. The scattering properties of the dermal layers are predominantly attributed to the fibrous structures of this tissue, where collagen fibrils are densely packed into collagen bundles and exhibit a lamellar arrangement.^23^ Due to the absence of melanin in this layer, significant variations in absorption across different skin phototypes are not observed. However, the thickness of the dermis also varies across different body regions. It is thickest on the back (up to 4 mm) and thinnest on the eyelids (approximately 215  μm),^1^ with an average thickness typically ranging between 1 and 4 mm.^5^

### 2.3 Subcutaneous Adipose Tissue Optical Properties: Role of Lipids, Water, and Hemoglobin

Subcutaneous adipose tissues are characterized by aggregations of fat cells, predominantly adipocytes, which store lipids in the form of small droplets. Blood capillaries, nerves, and reticular fibrils are interspersed within the spaces between these cells. The absorption coefficient of adipose tissue is primarily influenced by the absorption of hemoglobin, water, and lipids (with a characteristic peak around 930 nm), while the main scatterers are the uniformly distributed small lipid droplets within this layer.^30^^,^^31^ The thickness of the subcutaneous layer is highly dependent on an individual’s body fat composition, but on average, it ranges from 1 to 6 mm, depending on the specific body site.^5^

## 3 Methodologies for Determining Tissue Optical Properties: Challenges and Variability

The determination of tissue optical properties is a complex endeavor, with various methodologies yielding results that can exhibit significant variability. Understanding these methods and their inherent limitations is crucial for interpreting reported values.

### 3.1 Analytical Models versus Experimental Measurements

Two primary approaches are employed to assess the optical coefficients of tissues:

#### 3.1.1 Analytical models

These models derive optical properties based on the known biochemical composition of a particular tissue layer. They typically involve a baseline absorption and scattering, to which the relative contributions of the main absorbers (chromophores) and scatterers within the layer are added.^3^ The accuracy of this method is heavily reliant on: i) the precise estimation of the concentrations of the chromophores and scatterers considered, and ii) the precise knowledge of the absorption and/or scattering properties for each particle types. Although providing a theoretical framework, these models are limited by the completeness and accuracy of the underlying compositional data.

#### 3.1.2 Experimental measurements

These involve direct measurements on tissue samples, either *in vivo* (on living subjects) or *ex vivo* (on excised tissues). A typical experimental setup for *ex vivo* measurements involves carefully separating skin tissue layers and placing them between integrating spheres to measure diffuse transmittance and reflectance.^4^ These measured values are then frequently used in conjunction with inverse Monte Carlo methods or diffusion theory to calculate the absorption and reduced scattering coefficients.

### 3.2 Sources of Variability in Experimental Measurements

Despite advancements in measurement techniques, experimental determinations of the tissue optical properties are subject to considerable variabilities. This dispersion is particularly pronounced for *in vivo* measurements^1^ and, to a lesser extent, for *ex vivo* measurements.^2^^,^^10^

Key factors contributing to these variabilities include^2^^,^^10^: sample preparation, storage conditions and time, time between excision and measurement, measurement method itself, inverse problem-solving algorithm, tissue type and inter-individual variability, and biological state: (blood flow and oxygenation). It should be noted that the key factors mentioned above are not exhaustive. Indeed, factors such as physical stress on tissue during measurements and tissue hydration may also impact the tissue optical properties.^32^

These numerous contributing factors underscore the challenge of obtaining universally consistent and reliable optical property values, necessitating a critical interpretation and analysis of the existing literature.

## 4 Results: Absorption and Reduced Scattering Coefficients (*μ_a_* and $\mu_{s}^{\prime }$) of the Human Skin and Muscle

This section reviews reported values of $\mu_{a}$ and $\mu_{s}^{\prime }$ measured *ex vivo* for the three primary skin layers (epidermis, dermis, and subcutaneous fat) and muscle tissue at wavelengths relevant in photomedicine, namely: 454, 525, 594, 627, 729, and 842 nm. The literature includes both theoretical estimations, or mixed approaches, as performed by researchers such as,^21^^,^^22^^,^^33^^–^^35^ and experimental data measured on freshly excised samples.^4^^,^^5^^,^^16^^–^^20^^,^^36^ All datasets compiled here to illustrate the strategy we propose to derive a unique set of optical coefficients from the literature correspond to the “Caucasian” phototype as it is well reported in the literature, with the exception of,^36^ which considers Asian phototypes; for this reference, only data for the dermis and fat layers are considered due to potential epidermal differences. The number of available datasets varies across different wavelengths, as not all references provide results for every wavelength of interest. Furthermore, for the purpose of defining parameter sets for subsequent MC simulations as described below in Sec. 4.1, only references reporting both $\mu_{a}$ and $\mu_{s}^{\prime }$ values were considered.

### 4.1 *Ex Vivo* Measurements of the Human Skin Layers (Epidermis, Dermis, Subcutaneous Fat)

Following a comprehensive comparison of all available data, an initial pre-selection process was conducted, leading to the rejection of references^33^^–^^35^ due to their reporting of outlier points^37^ compared with others. The selected values of $\mu_{a}$ and $\mu_{s}^{\prime }$ are shown in Fig. 2. As expected, both coefficients tend to exhibit a general decrease with increasing wavelength. This effect is particularly pronounced around 454 and 525 nm, while becoming less prominent around 730 and 840 nm. It is also notable that values at shorter wavelengths tend to show greater relative and absolute dispersions, likely attributable to the strong influence of hemoglobin absorption in these spectral regions.

**Fig. 2:**
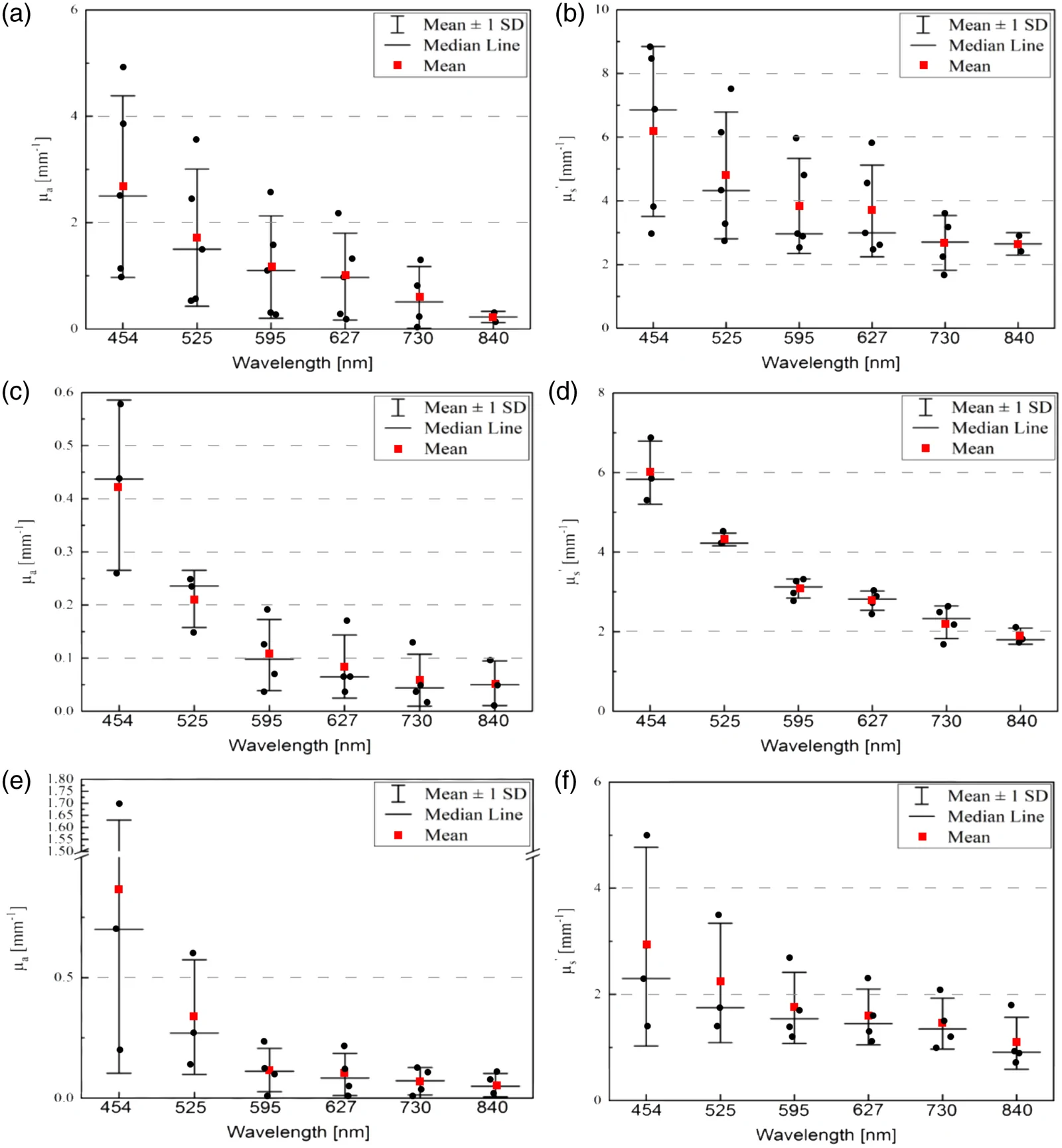
Reported values of absorption $\mu_{a}$ and reduced scattering $\mu_{s}^{\prime }$ coefficients at different wavelengths. Epidermis (a)–(b), dermis (c)–(d), and subcutaneous adipose tissue (e)–(f), across wavelengths from 454 to 840 nm. Left panels show $\mu_{a}$ and right panels show $\mu_{s}^{\prime }$. For each wavelength, the mean ± SD, median, and individual reported values are displayed. The data illustrate the general decrease in both $\mu_{a}$ and $\mu_{s}$ with increasing wavelength, alongside substantial variability in published measurements. Particularly at shorter wavelengths and in layers with higher chromophore content.

To identify the sets of optical properties for MC simulations for each layer and at each wavelength of interest, a new procedure was adopted. This approach consists of determining the median, first, and third quartiles of the effective attenuation coefficient $\left(\mu_{\mathrm{eff}}=(3\;\mu_{a}(\mu_{a}+\mu_{s}^{\prime }){)}^{-1/2}\right)$ and the transport albedo $\left(a^{\prime }=\mu_{s}^{\prime }/\mu_{a}+\mu_{s}^{\prime }\right)$^38^ from a statistical ensemble of 3 to 5 pairs of $\left(\mu_{a};\mu_{s}^{\prime }\right)$ for each condition. We selected the effective attenuation coefficient and the transport albedo, which are both depending on $\mu_{a}$ and $\mu_{s}^{\prime }$ only, because they reflect the sample macroscopic optical properties determining, respectively, the depth of light penetration in biological tissues and their diffuse reflectance.^8^ In addition, note that inter-study relative fluctuations in $\mu_{a}$ and $\mu_{s}^{\prime }$ are usually larger than those in $\mu_{\mathrm{eff}}$ or $a^{\prime }$ because $\mu_{a}$ and $\mu_{s}^{\prime }$ are often inferred jointly from coupled theoretical models.^2^^,^^36^^,^^38^^,^^39^ For example, both $\mu_{\mathrm{eff}}$ and reflectance values depend on $\mu_{a}$ and $\mu_{s}^{\prime }$. Therefore, an overestimate of $\mu_{s}^{\prime }$ typically produces a very significant underestimate of $\mu_{a}$, and vice versa.

Figure 3 presents an illustrative map showing iso-curves of $\mu_{\mathrm{eff}}$ and $a^{\prime }$ (median also described as second quartile, first, and third quartile values) for the three skin layers at 840 nm. These iso-curves intersect at specific pairs values of $\mu_{a}$ and $\mu_{s}^{\prime }$. The intersection of $\mu_{\mathrm{eff}}$ and $a^{\prime }$ median lines defines our estimated values of a unique set of $\mu_{a}$ and $\mu_{s}$, an operation we performed for all layers and wavelengths. In the same way, the four intersection points of the first and third quartile curves provide extreme pairs of $\mu_{a}$ and $\mu_{s}^{\prime }$, which can be utilized to evaluate variations in light propagation and define error margins in the assessment of light fluence.

**Fig. 3:**
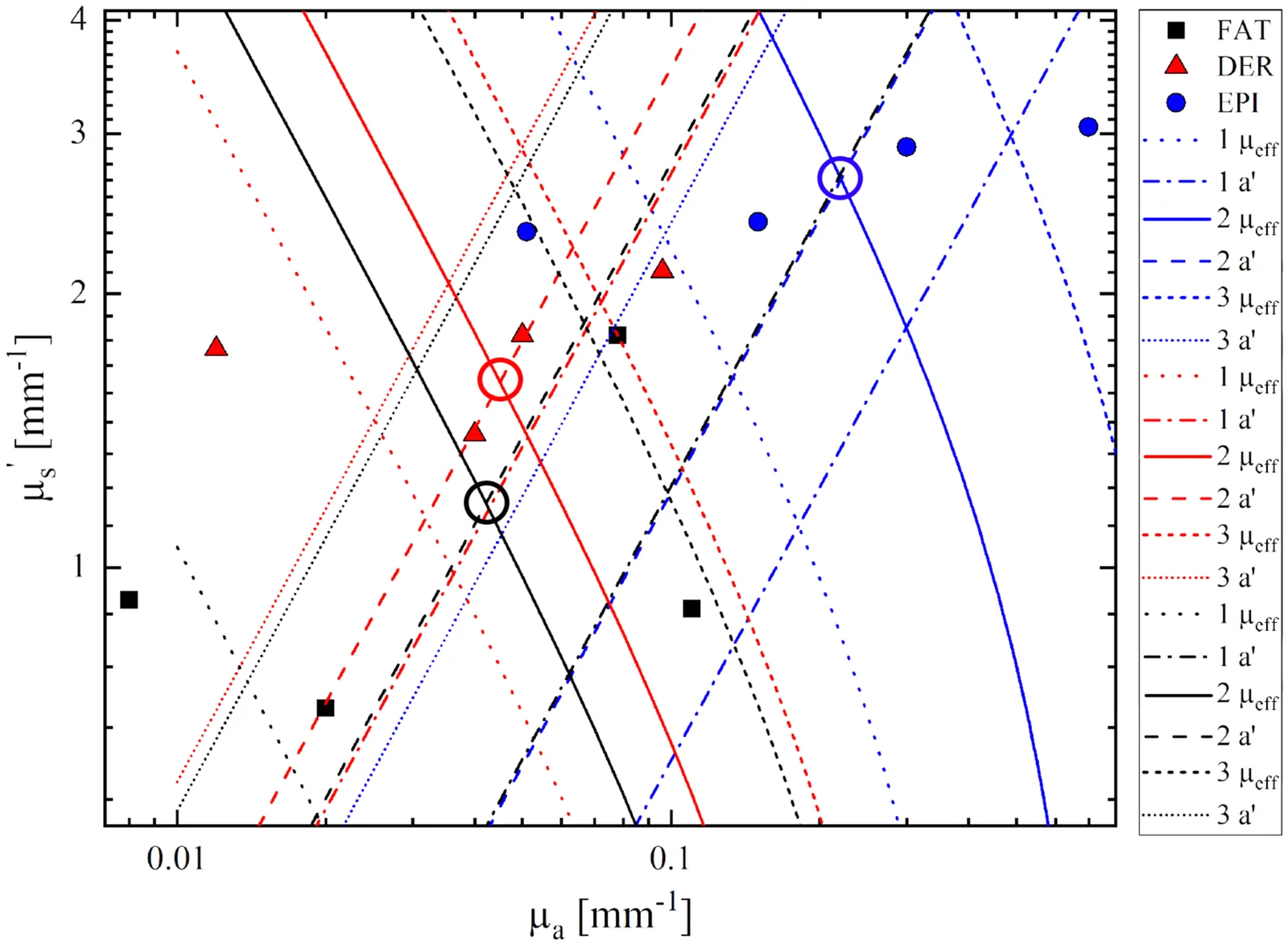
Statistical map of $\mu_{a}$ and $\mu_{s}^{\prime }$ for the epidermis (EPI; blue lines), dermis (DER; red lines), and fat tissues (FAT; black lines) at 840 nm. This figure compiles literature-reported pairs of absorption $\mu_{a}$ and reduced scattering $\mu_{s}^{\prime }$ coefficients (squares) for the fat, dermis, and epidermis. The plot demonstrates the methodology used to derive a representative set of optical properties: $1\;\mu_{\mathrm{eff}}$, $2\;\mu_{\mathrm{eff}}$ and $3\;\mu_{\mathrm{eff}}$ stand for the first quartile, median and third quartile values of the effective attenuation coefficient $\mu_{\mathrm{eff}}$, respectively. The same labeling is used for the transport albedo $a^{\prime }$. The intersections of the median iso-curves for $\mu_{\mathrm{eff}}$ and $a^{\prime }$ define the median pair ($\mu_{a}$ $\mu_{s}^{\prime }$) representative of the optical coefficients. The intersections of the first and third quartile curves define the extremes, which can be used to evaluate uncertainty in simulations.

Figure 4 presents only the calculated median values of $\mu_{a}$ and $\mu_{s}^{\prime }$ for all wavelengths and tissue layers. These values are compiled in Table 1.

**Fig. 4:**
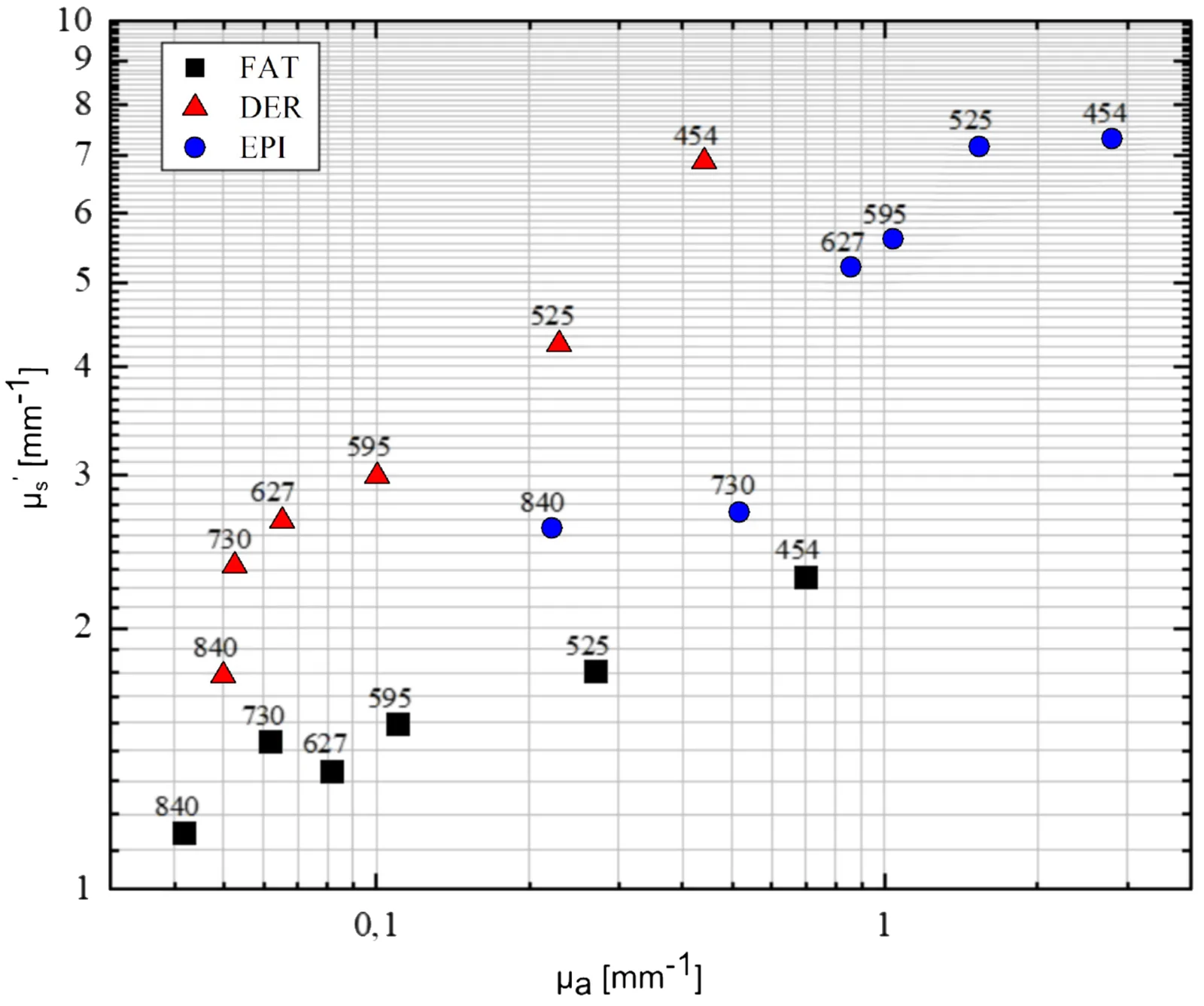
Summary of the optical coefficients corresponding to the intersection of the median values of $\mu_{\mathrm{eff}}$ and $a^{\prime }$ pairs used for Monte Carlo simulations. This map displays the absorption ($\mu_{a}$) and reduced scattering coefficients ($\mu_{s}^{\prime }$) corresponding to the median values of $\mu_{\mathrm{eff}}$ and $a^{\prime }$ calculated for each tissue layer. The data points are color-coded by tissue layer (epidermis: EPI, dermis: DER, subcutaneous adipose: FAT) and labeled with their corresponding wavelength. This figure provides a visual summary of the derived optical parameters, which can be used for simulations.

**Table 1:** Optical parameters for Monte Carlo simulations. The table reports the “representative” values for the absorption $\mu_{a}$ and reduced scattering $\mu_{s}^{\prime }$ coefficients, scattering anisotropy factor g, and refractive index n selected for each of the four layers: epidermis (EPI), dermis (DER), subcutaneous adipose (FAT), and muscle (MUS). These values are derived from the critical literature analysis presented in this review.

	Thickness (mm)	λ (nm)	n	g	$u_{a}$ (${\mathrm{mm}}^{-1}$)	$u_{s}^{\prime }$ (${\mathrm{mm}}^{-1}$)	$u_{s}$ (${\mathrm{mm}}^{-1}$)
EPI	0.075 to 0.150	454	1.45	0.73	2.81	7.29	27.0
		525	1.445	0.75	1.53	7.16	28.6
		595	1.44	0.77	1.04	5.58	24.3
		627	1.435	0.78	0.86	5.17	23.5
		730	1.43	0.81	0.52	2.72	14.3
		840	1.42	0.84	0.22	2.40	15.0
DER	1 to 4	454	1.39	0.57	0.44	6.87	16.0
		525	1.38	0.65	0.23	4.27	12.2
		595	1.379	0.70	0.10	3.01	10.0
		627	1.378	0.72	0.065	2.67	9.5
		730	1.373	0.78	0.053	2.41	11.0
		840	1.375	0.81	0.05	1.77	9.5
FAT	1 to 6	454	1.47	0.90	0.70	2.29	22.9
		525	1.467	0.90	0.27	1.78	17.8
		595	1.463	0.90	0.11	1.55	15.5
		627	1.461	0.90	0.082	1.37	13.7
		730	1.458	0.90	0.062	1.42	14.8
		840	1.456	0.90	0.042	1.16	11.6
MUS	Semi-infinite	454	1.41	0.87	0.97	1.8	13.8
		525	1.39	0.90	0.65	1.26	12.6
		595	1.379	0.92	0.31	0.97	12.1
		627	1.378	0.92	0.23	0.87	10.9
		730	1.373	0.93	0.10	0.67	9.6
		840	1.37	0.93	0.085	0.57	8.15

### 4.2 Optical Properties of Muscle Tissue

Muscle tissues are typically located directly beneath the subcutaneous adipose layer in most body sites. Because the thickness of muscle is much larger than $\mu_{\mathrm{eff}}^{-1}$ at many anatomic locations, it is frequently considered as a “last” layer with infinite thickness in light propagation models.^40^ Although only a small fraction of photons transmitted through the air-skin interface are likely to reach this muscle layer, particularly for shorter (<580  nm) wavelengths illuminating thick cutaneous tissues, its inclusion in simulations is necessary to avoid artifacts resulting from edge effects at the lowest skin interface.

A simplified procedure can be employed to determine $\mu_{a}$ and $\mu_{s}^{\prime }$ for muscle at the wavelengths of interest due to two main reasons: 1) limited published data exist concerning the optical coefficients of human muscle at these specific wavelengths; and 2) *ex vivo* measurements, predominantly performed on animal muscles, have exhibited significant intra- (between different muscles) and inter-species variations in $\mu_{a}$ and $\mu_{s}^{\prime }$ (as can be seen in Fig. 5). This variability is, at least in part, attributed to factors such as muscle fiber orientation,^41^ as well as variations in water and hemoglobin content (the latter presenting different absorption properties depending on its redox state).^39^^,^^42^^,^^43^ Therefore, a decision was made to attribute $\mu_{a}$ and $\mu_{s}^{\prime }$ values to the muscle layer derived from a recent study based on measurements of fresh excised human tissue obtained from five cadavers,^44^ covering a spectral range between 350 and 2500 nm (represented by the black line in Fig. 5). It should also be noted that these data published by Haugen et al.^44^ are compatible with the values published in the reports mentioned above.^39^^,^^42^^,^^43^

**Fig. 5:**
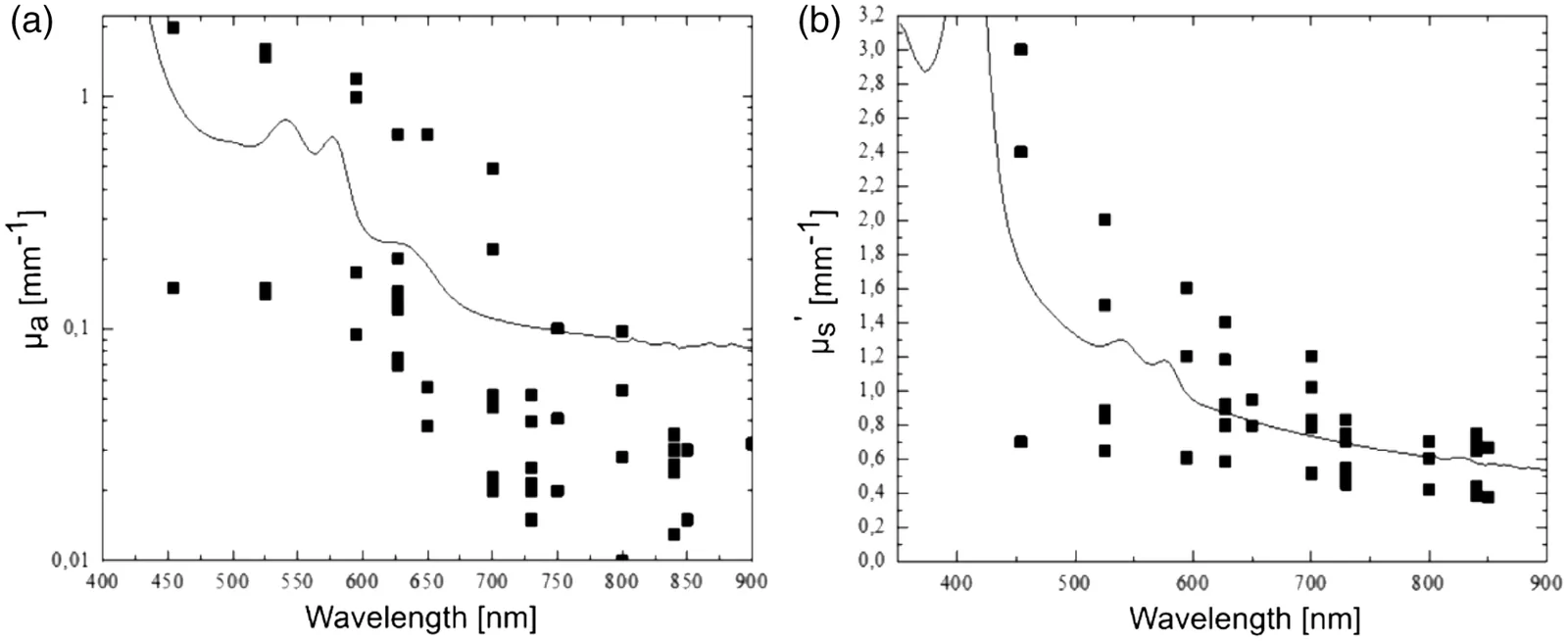
Comparison of published optical properties for muscle, highlighting the values selected for simulation. (a) The absorption coefficient ($\mu_{a}$) and (b) the reduced scattering coefficient ($\mu_{s}^{\prime }$) for muscle tissue as a function of wavelength. Black data squares represent values from a compilation of literature sources. The solid black line indicates the values reported by Haugen et al.^44^

## 5 Light Scattering Anisotropy Factor (*g*)

The light scattering anisotropy factor, g, such as other optical coefficients, is dependent on tissue type and wavelength.^45^ Typical reported *ex vivo* values at 630 nm are ∼0.72 for the dermis, 0.9 for muscle, and 0.97 for blood.^46^^,^^47^ In many MC simulations, g is frequently assumed to be constant across different tissue layers, with values typically ranging between 0.8 and 0.95 for red and near-infrared (NIR) light. However, it has been demonstrated that this approximation can introduce errors only on the order of several percent in the determination of the light fluence.^36^ The situation becomes more complex at shorter wavelengths, where values as low as 0.38 have been reported at 450 nm.^48^ This suggests the necessity of defining wavelength-specific g values for accurate MC simulations.

Given the scarcity of reliable experimental determinations of g, primarily due to the inherent difficulties in obtaining relevant values, an effective strategy involves the utilization of analytical models. The approach proposed by Jacques et al., which demonstrates excellent correlation with experimental data, is based on the following parametric function:^3^^,^^49^

$$g(\lambda )=A+B(1-\exp((\frac{\lambda -C}{D})),$$ (1)

where A, B, C and D are empirical factors, and λ is the wavelength. The factors A, B, C and D have no physical significances. For the subcutaneous adipose tissue, the only available values of g are such that it is generally assumed to be constant at 0.9.^20^ Figure 6 presents plots of *g* based on Eq. (1), with A, B, C, D parameters selected from Refs. 48,50,51 for the epidermis, the dermis, and the muscle layers, according to Ref. 5. It is interesting to note the evolution of g between “smooth” and “rough” porcine dermis samples (dashed line in Fig. 6), where “rough” samples show a higher isotropic behavior than “smooth” samples. Considering that the interfaces between different skin layers are inherently “rough,” values corresponding to the “rough” case are generally more appropriated for simulations.^5^

**Fig. 6:**
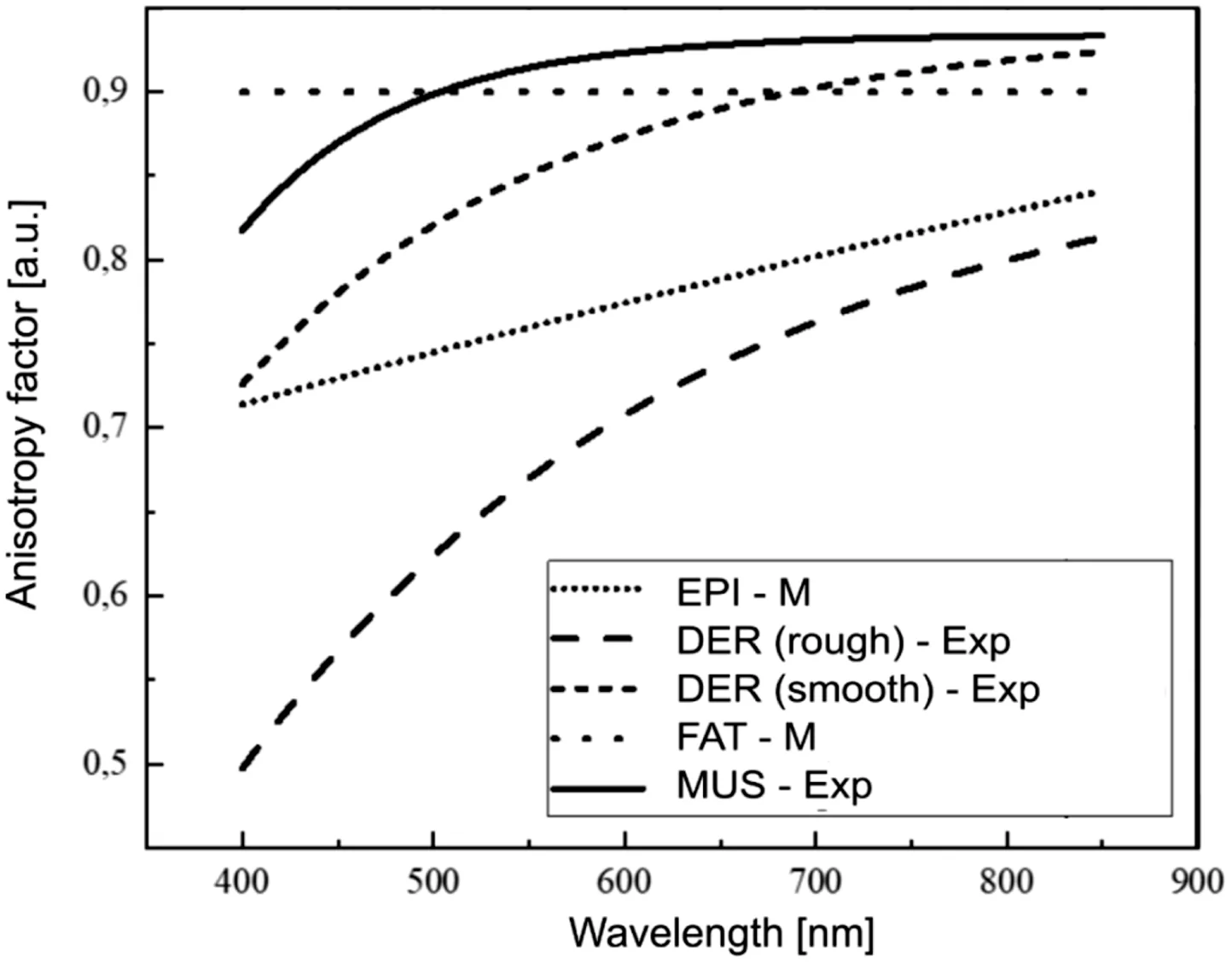
Light Scattering Anisotropy Factor g Spectra for human tissue layers. This figure presents the anisotropy factor as a function of the wavelength for the epidermis (EPI),^50^ dermis (DER),^48^ subcutaneous adipose (FAT),^49^ and muscle (MUS).^51^ The data are derived from either analytical models (M) or experimental measurements (Exp), with the parameters for each model listed below. The “rough” dermis model was selected to better represent the irregular tissue interfaces. Model parameters:^5^ EPI: A=0.745; B=0.546; C=500; D=1806; DER (rough): A=0.653; B=0.219; C=530.2; D=242.8; DER (smooth): A=0.84; B=0.099; C=530.2; D=170; MUS: A=0.883; B=0.051; C=469.3; D=84.11.

## 6 Refractive Index (*n*)

As a general rule, the refractive index n of biological tissues is linearly dependent on tissue density.^21^ With the exception of the human stratum corneum, which has an n value of approximately 1.55 in the visible part of the spectrum,^52^ it is widely accepted that, for most biological media, n typically ranges between 1.35 and 1.45, values slightly larger than that of water (n=1.33), which is the primary constituent of tissue. It is also generally accepted that n exhibits a weak wavelength dependence in the visible part of the spectrum. For this reason, similar to the anisotropy factor g, a constant value of n is often considered for a given tissue layer in many Monte Carlo simulations.^2^

However, it is important to note that n is influenced by tissue hydration^53^ and can show relatively significant variations across different tissues. For instance, n is ∼1.37 for muscle, whereas its value reaches about 1.44 for subcutaneous adipose tissue in the visible part of the spectrum.^54^ Therefore, to accurately account for refractive index differences at layers interfaces, we propose to attribute specific wavelength-dependent values of n for MC simulations. The Cauchy dispersion parametric functions can be used^27^^,^^55^^,^^56^ which writes

$$n(\lambda )=A+B\lambda^{-2}+C\lambda^{-4},$$ (2)

where A, B, and C are empirical factors. Figure 7 presents n based on Eq. (2) with A, B, C parameters for the skin layers, derived from Caucasian post-mortem human samples.^27^^,^^57^

**Fig. 7:**
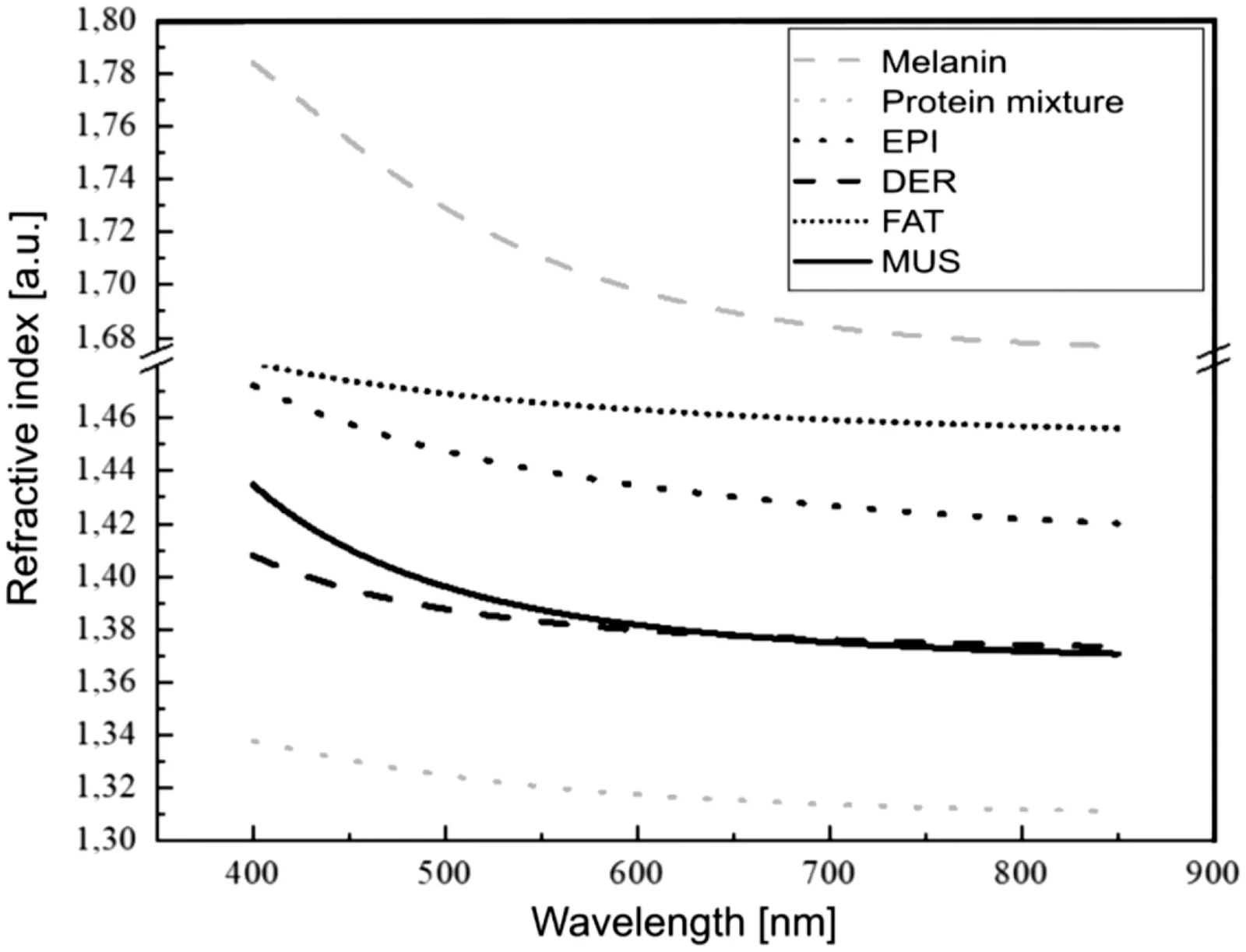
Refractive index (n) spectra of the four-layer tissue model. The figure presents the wavelength-dependent refractive index for the epidermis (**EPI**),^27^ dermis (**DER**),^27^ subcutaneous adipose (**FAT**),^57^ and muscle (**MUS**).^55^ These values were determined using the Cauchy dispersion parametric function with the specific coefficients listed below, which were derived from literature^27^^,^^57^ for Caucasian tissue samples; **EPI:** A=1.46; $B=10^{4}$; $C=10^{8}$; **DER:** A=1.37; B=1512; $C=7\times 10^{8}$; **FAT:** A=1.449; B=5099; C=0; **MUS:** A=1.365; B=1500; $C=15\times 10^{8}$.

## 7 Discussion and Future Perspectives

The accurate determination and compilation of tissue optical properties are foundational for reliable Monte Carlo simulations of light propagation in biological media. As this review highlights, the optical landscape of most human tissues, including the skin and underlying muscle, is complex, characterized by distinct layered structures, varying chromophore concentrations, and diverse scattering elements. Although progresses are made to improve the accuracy of the measured skin optical coefficients,^25^ the critical analysis of existing literature underscores a significant challenge: the considerable variability in reported optical coefficients. This dispersion is not merely an inconvenience but frequently a critical factor that can introduce substantial uncertainties in light dosimetry predictions, impacting the efficacy and safety of photo-medical applications. It is indeed reasonable to expect that modelling the propagation of light using optical coefficients that are less prone to fluctuations and therefore determined with greater accuracy and precision, as noted above for the approach proposed in this article, will yield more reliable results. A quantitative assessment of the improvement for specific configurations (e.g., particular optical coefficients and illumination geometries) must be addressed on a case-by-case basis and will be the subject of a separate studies.

The observed variability stems from a confluence of factors, including inter-individual differences, variations in measurement methodologies, sample preparation, uneven or unsharp interfaces between different layers, and the dynamic physiological state of *in vivo* tissues. While theoretical models provide valuable insights into the biophysical basis of optical properties, experimental measurements, particularly *ex vivo*, offer more direct data. However, even *ex vivo* data are susceptible to changes post-excision and depend on methodological biases.

The new and systematic approach we propose here, which is based on analyzing median and quartiles of the effective attenuation coefficient, and the transport albedo, represents a relevant strategy for deriving unique sets of representative or consensus optical parameters from disparate datasets. This approach, which can be adapted for the optical parameters of other tissues but the skin, also offers a consistent mean to quantify uncertainty. Like all methods for deriving optical coefficients, ours requires a sufficiently large dataset; however, for the reasons given above, the dataset needed to reach a given level of reliability is smaller than that required by the standard approach of estimating the mean of each optical coefficient. A quantitative assessment of the dataset size required to achieve a specified reliability for particular cases will be presented in a separate study.

The compilation and determination of typical values of $\mu_{a}$ $\mu_{s}^{\prime }$ g, and n for phototype III epidermis, dermis, subcutaneous fat, and muscle, as presented in this review as an illustrative example, provides a valuable resource for researchers developing and utilizing MC simulations. The emphasis on a multilayered skin model is crucial, as a homogeneous single-layer approximation fails to capture the intricate light-tissue interactions occurring, especially within the superficial layers.^6^^,^^58^ The wavelength dependence of these properties, mostly resulting from the strong influence of hemoglobin at wavelengths shorter than 600 nm, and the need for wavelength-specific g and n values, further highlights the complexity.

Looking forward, several key areas warrant further research to enhance the accuracy and reliability of tissue optical property data:

**Standardization of measurement protocols:** Developing universally accepted protocols for *ex vivo* and *in vivo* optical property measurements would significantly reduce inter-study variability and improve data comparability and fluctuations.

**Advanced *in vivo* techniques:** Continued development of noninvasive *in vivo* measurement techniques that can accurately quantify optical properties while accounting for physiological dynamics (e.g., blood flow, oxygenation, and hydration) is paramount.

**Comprehensive databases:** Establishing and maintaining publicly accessible, curated databases of tissue optical properties, completed with detailed metadata on measurement conditions, tissue source, and analytical methods, would be invaluable.

**Better taking into account of the stratum corneum:** Although this skin layer is frequently neglected due to its small thickness, its relatively large refractive index combined with the microstructures/roughness of its surface and interface may impact significantly the propagation of light in certain situations. It should be noted that the integration of this skin layer in MC simulations and the application of the strategy proposed in this article to derive a unique set of optical parameters is nevertheless not problematic once these data will be available.

**Dynamic optical properties:** Research into how optical properties change in real-time due to physiological responses (e.g., vasodilation, inflammation, and drug administration) is essential for dynamic light dosimetry.

**Validation of analytical models:** Further validation and refinement of analytical models for *g* and *n* against a broader range of experimental data are needed, especially for less studied tissue types and wavelengths.

Ultimately, improvements in the precision and consistency of tissue optical property data will directly translate to more accurate MC simulations, leading to better-informed decisions in the design and application of light-based medical technologies.

As mentioned above, only the skin phototype III was considered in this illustrative study to identify a unique set of optical parameters to be used to model the propagation of light in the skin with Monte Carlo simulations at one wavelength. Nevertheless, the approach proposed here to derive these parameters from the literature can easily be adapted for darker or lighter phototypes. Indeed, several groups have published values of the skin optical properties for different phototypes.^59^^–^^62^ It should be noted that, for normal skin, the epidermis is the only layer in which the optical properties are changed for different skin phototypes due to its different contents of melanin^60^ a pigment that is responsible for a monotonic decrease of µ_a_ for increasing wavelenghs.^1^

Another question of interest consists to determine if the reported values of the skin layers optical coefficients obtained *in vivo* are more or less reliable as compared to those measured *ex vivo*, although *ex vivo* samples contain less blood as compared to the *in vivo* situation. The advantages and drawbacks of both approaches have been reported in details by Setchfield et al.^1^ In general, the *ex vivo* and *in vivo* values of $\mu_{a}$ and $\mu_{s}^{\prime }$ show similar trends. However, this group reported that the average *in vivo* values of $\mu_{s}^{\prime }$ beyond 480 nm are approximately 1.7-fold less than in the *ex vivo* situation, whereas this difference decreases to 1.4-fold between 800 and 1300 nm. In this last spectral range this group also reported that the *ex vivo* values of $\mu_{a}$ are approximately 1.6-fold greater than those obtained *in vivo* (compared with a 2.5-fold average difference across the whole spectrum). It should be noted that, in general, the variations of the values reported in the literature for $\mu_{a}$ and $\mu_{s}^{\prime }$ are much larger than the differences mentioned above when comparing *in vivo* and *ex vivo* measurements. This statement, together with other measurements of the optical properties we performed on the same tissue samples *in vivo* and postmortem in a separate study, strongly suggests that optical coefficients in the red and NIR are not substantially altered between *in vivo* and *ex vivo* conditions, provided the samples have not been frozen or fixed in formalin.^10^^,^^63^ The implementation in MC simulations of the biological tissues optical coefficients resulting from the literature analysis and data processing presented in this article is generally straightforward since the vast majority of these programs require the direct entry of a unique set of these coefficients as input parameters.^64^ As noted above, our method for determining these unique parameter sets relies on selecting $\mu_{\mathrm{eff}}$ and $a^{\prime }$, both of which depend on $\mu_{a}$ and $\mu_{s}^{\prime }$. This choice was not only made because $\mu_{\mathrm{eff}}$ and $a^{\prime }$ capture the sample’s macroscopic optical behavior, but also because many techniques for estimating $\mu_{a}$ and $\mu_{s}^{\prime }$ treat these two last parameters as coupled. Consequently, overestimating $\mu_{a}$ often corresponds to underestimating $\mu_{s}^{\prime }$, and vice versa, so computing simple averages of reported $\mu_{a}$ and $\mu_{s}^{\prime }$ values is suboptimal.

MC simulations may account for more than three layers, according to the geometry of the skin model considered. This is, in particular, the case for the Monte Carlo modeling of light transport in multilayered tissues (MCML) reported by Wang et al.^58^ or the seven- or nine-layer skin models reported by Maeda et al.^6^ It is obvious that the strategy presented here to derive a unique set of optical coefficients from the literature can be applied for more than three layers. However, although very few data are available for large numbers of skin layers, we decided to illustrate our strategy with a three-layer model.

Finally, it should be noted that the unique set of optical coefficients obtained with our novel approach, based on the intersections of the curves corresponding to the medians of the effective attenuation coefficient $\mu_{\mathrm{eff}}$ and the transport albedo $a^{\prime }$, is consistent with those reported in seminal articles in the field.^8^^,^^9^ Indeed, most of the values of the absorption and scattering coefficients we obtained lie between those reported by Meada et al. and Finlayson et al.^6^^,^^7^ who published a compilation of optical coefficients derived from the literature. In addition, our values rarely differ by more than 50% from those found in these reports, and the spectral trends are similar. This compatibility with the optical-parameter values reported by other groups studying multilayer skin models supports the validation of this new approach.

## 8 Conclusion

This article not only compiles and critically evaluates the variations of the reported optical coefficients for human skin (Phototype III) and muscle, as an illustrative example, for selected visible and near-infrared wavelengths, but it also proposes a new approach to derive unique sets of these coefficients for different skin layers. This review has systematically examined the optical properties of human skin (Phototype III) and underlying muscle tissue, essential parameters for accurate Monte Carlo simulations of light propagation, which require a unique set of optical coefficients per tissue layer and wavelength. The layered structure of the skin, comprising the epidermis, dermis, and subcutaneous fat, each with distinct chromophores and scatterers, necessitates a multilayered modeling approach. Although theoretical models provide a foundational understanding, experimental measurements, despite their inherent variability, remain crucial for practical applications.

The compilation and critical analysis of absorption coefficients $\mu_{a}$, reduced scattering coefficients $\mu_{s}^{\prime }$, anisotropy factors g, and refractive indices n reveal a complex interplay of wavelength dependence, tissue composition, and methodological influences. The selected “typical” values for $\mu_{a}$ and $\mu_{s}^{\prime }$ for each layer and wavelength, derived through an approach based on the values of $\mu_{\mathrm{eff}}$ and $a^{\prime }$, provide a valuable dataset for light dosimetry studies. Similarly, the use of analytical parametric functions for g and n ensures wavelength-specific and tissue-specific considerations. The inclusion of muscle optical properties addresses potential edge effects in deeper tissue simulations.

## Data Availability

No custom code was developed for this study. The data supporting the findings of this work were obtained from previously published literature sources, all of which are cited in the paper. The extracted and processed data used for the analyses presented in this article are available from the corresponding author upon reasonable request.
